# Children’s Sense of Belonging in the Context of Post-separation Parental Stalking: Finnish Children’s Experiences of their Family Relations

**DOI:** 10.1007/s40653-022-00494-x

**Published:** 2022-11-18

**Authors:** Anna Nikupeteri, Merja Laitinen, Kati Kallinen

**Affiliations:** 1 Faculty of Social Sciences, University of Lapland, Yliopistonkatu 8, 96300 Rovaniemi, Finland; 2 Department of Social Sciences, University of Eastern Finland, Kuopio, Finland

**Keywords:** Stalking, Domestic violence, Significant relationships, Child’s best interest, Children’s safety

## Abstract

Drawing from previous research on domestic violence and stalking, this study addresses children’s experiences of their family relations in post-separation parental stalking contexts from the perspective of stalking as a form of violence against women and children. Despite the fact that violence perpetrated by a parent fundamentally changes family dynamics and children’s perceptions of family security, research on children’s family relations in the course of domestic violence or stalking has rarely addressed children’s sense of belonging. The aim of this paper is to augment our understanding of children’s experiences of family relations in connection with parental stalking. The research question is: How do children experience their belonging in family relations in the context of post-separation parental stalking? A total of 31 children and young people aged 2–21 participated in the study. The data were collected through interviews and therapeutic action group sessions with the children. The qualitative data analysis was content-oriented. Four dimensions of children’s sense of belonging were identified: (1) Varying belonging*,* (2) distancing belonging, (3) non-belonging and (4) holding belonging. The first three dimensions are constructed in relation to the child’s stalking father, while the fourth one includes the mother, siblings and other relationships that provide safety and comfort. The dimensions are parallel and not mutually exclusive. The study indicates that a finer-grained understanding of children’s sense of belonging in family relations is needed when social and health care and law enforcement professionals evaluate the child’s safety and best interest.

“Do I have to love my father if I don’t want to?” This is a question asked by one of the child interviewees from the mother during our research project^1^ – and one that prompted us to consider children’s sense of belonging in the context of post-separation parental stalking. The child’s question is reflective of the Western cultural ethos in post-separation situations: joint parenting, both parents’ contribution to the child’s upbringing during post-separation, and the perception of the abusive partner as a capable father (e.g., Elizabeth et al., 2012; Holt, 2015; Jaffe et al., 2009; Radford & Hester, 2015). This way of thinking may, indeed, disregard parental violence and thereby undermine children’s – and their mothers’ – wellbeing and security in cases where violence and stalking continue after parental separation.

In this paper, post-separation parental stalking is connected to previous research on domestic violence and post-separation violence (e.g., Callaghan et al., 2015; Cater, 2007; Cater & Forssell, 2014; Katz, 2015; Noble‐Carr et al., 2020) and stalking is approached as a form of violence against women and children (e.g., Logan & Walker, 2017). Studies have shown that when women leave their abusive partner, it does not always end the violence; on the contrary, it may intensify in the aftermath of the separation or continue as different forms of violence, coercive control, and stalking (Bendlin & Sheridan, 2021; DeKeseredy et al., 2017). Although women are statistically more often victims of stalking than men in different samples (e.g., Spitzberg & Cupach, 2014), women can also be perpetrators and mothers can target stalking behavior at the child’s father (e.g., Brooks et al., 2021), and stalking can appear in same-gender relationships as well (Strand & McEwan, 2011).

In this study, the stalking is defined as intense and pursuit-oriented action with a pattern of repeated, intrusive, and intentional behaviors – such as following, harassing, and threatening – that cause fear and distress in victims (e.g., Chan & Sheridan, 2020; Logan & Walker, 2017; Spitzberg & Cupach, 2014). Parental stalking can include violent and non-violent acts. On the one hand, it may include intimidation, exploitation of the children in controlling the mother, physical abuse and threats of death. On the other hand, it may consist of the father’s performance of care, love and longing for the children (Nikupeteri & Laitinen, 2015; Løkkegaard et al., 2019). It has negative socioemotional and psychosocial consequences for children that become visible in the relationships with their parents. The consequences are exacerbated by children’s memories of their history with the abusive parent and by their present life, which is marked by negotiations between the mother and father and attempts to deal with the stalking. Stalking as a form of domestic violence undermines children’s agency and wellbeing and limits their possibilities to act and seek help (Nikupeteri & Laitinen, 2015; Elklit et al., 2019; Logan & Walker, 2017; Pang & Thomas, 2020).

Studies show that where the father has been abusive towards the mother and/or the children, the children’s relationship with the father is complicated and they may hold ambivalent views of him (e.g., Arai et al., 2021; Holt, 2015; Øverlien, 2013). In several previous studies, children have described their relationship with the father as being characterized by fear, hate, anger and disappointment, but also by warmth, kindness, love and care for him (e.g., Cater, 2007; Mullender et al., 2002; Staf & Almqvist, 2015). Other studies have shown that children perceive their abused mothers as their primary caregivers who provide protection, increase their wellbeing and promote their recovery (e.g., Cater & Forssell, 2014; Katz, 2015; Lapierre et al., 2018; Leung, 2015). A qualitative systematic review of children’s perspectives on domestic violence and abuse found that children respond strategically and in diverse ways to experienced abuse in their relationships, for example by protecting self and others (Arai et al., 2021). When experiencing violence in family relations, the child may lose both the sense of being cared for and nurtured and the sense of trust in the caregivers’ capacity to provide support and protection (Swanston et al., 2014). When children become aware of their habitual ways of being and doing and then notice that they cannot go about the mundane tasks that they are accustomed to, they may develop a feeling of not belonging and uneasiness in relating to family members, particularly to the abusive parent (see May, 2011).

This study is a part of a larger Finnish project Children's Knowing Agency in Private, Multiprofessional and Societal Settings - The Case of Parental Stalking investigating children’s experiences and agency in parental stalking. Based on earlier research on children exposed to domestic violence, the hypothesis was that children’s accounts can offer important knowledge on parental stalking. Drawing on children’s own accounts, it is possible to get new insights into ex-partner stalking and to create an understanding of parental stalking that can be utilized in developing services for children and in considering custody arrangements and child-parent contacts after parents’ separation. The present study thus focuses on the question of how children experience their belonging in family relations in the context of post-separation parental stalking. The question is addressed through the concept of *belonging*. Belonging is a contextual and multifaceted issue which can be affected by various factors and approached from different perspectives that include issues such as space, ethnicity and culture (May, 2011). The approach to belonging in this study is based on children’s family relations. The sense of belonging is regarded as being based on family relations that are constructed in practices of intimacy, which sustain a subjective sense of closeness and enable people to be attuned and special to each other (Jamieson, 2011). Children’s memories of violence and stalking perpetrated by a parent and the emotions arising from these experiences affect the way in which they engage in family relations. Belonging, as a relational concept involving social interaction and intersubjectivity, is much about the emotions emerging through this interaction (May, 2011). Thus, the sense of belonging is closely connected to Giddens’s (1991) theorisations of ontological security and pure relationships. The former enables people to experience fundamental safety by having basic trust in other people, to maintain psychological wellbeing and to avoid anxiety. The latter means that people can keep up relationships for their own sake.

Belonging also plays a role in constructing the sense of self in relation to more abstract notions of collectively held social norms and values (May, 2011). Children’s possibilities to maintain and create safe family relations after their parents’ separation are connected to the cultural and societal contexts. The ideal of joint parenting informs the work of professionals and underlies legal agreements, which may lead to a situation in which the child’s contact with the abusive parent is sustained even though it would be safer to avoid it from the child’s perspective (Bergman & Eriksson, 2018; Humphreys et al., 2019; Løkkegaard et al., 2019). Post-separation contact arrangements should recognise the complexity of children’s family relations (see Noble-Carr et al., 2020) and their personal views of how safe they feel in those relations.

The aim of this paper is to augment our understanding of children’s experiences of family relations in connection with parental stalking (e.g., Nikupeteri & Laitinen, 2015; Elklit et al., 2019; Løkkegaard et al., 2019) and, more broadly, in the context of domestic violence. Despite the fact that violence perpetrated by a parent fundamentally changes family dynamics and children’s perception of secure family relations (e.g., Mullender et al., 2002), the sense of belonging appears not to have been in the focus of research on children’s family relations in the contexts of domestic violence and stalking. We argue that in order to safeguard children’s safety and best interest when faced with post-separation parental stalking, actors within social and health care services and law enforcement need to develop a finer-grained understanding of children’s experiences of their sense of belonging in family relations.

## Methodology

### Participants and Data Collection

A total of 31 children and young people (23 girls, 8 boys) aged 2–21 participated in the study. They were all Finns, but in a few cases, the father had been born outside Finland. All the children lived with their mothers and almost everyone had one or more siblings. Some had been directly abused by the stalker, whereas others had been used as a means of their father’s or stepfather’s stalking of their mother. Most of the children were still experiencing stalking, although in three cases the stalking had ended because the stalker had deceased. In most instances, the stalker was the child’s biological father, and whether the stalker was the biological father or stepfather, there were no differences in how the children experienced the degree of harmfulness of the stalking. Therefore, we use the term “father” throughout the paper. As for some of the children, there was or had previously been a restraining order against the father. Many were in contact with their father, at least through supervised meetings.

Stalking as a dynamic and varied phenomenon affected the total sample. Children and mothers who are stalked feel pervasive fear, can be targets of death threats, undergo multiple relocations, and may even live ‘underground’ because of them (e.g., Nikupeteri & Laitinen, 2015; Logan & Walker, 2017). All things considered, the risk of harmful consequences caused by the children’s participation in the study had to be critically evaluated (Laitinen & Nikupeteri, 2018).

The children were recruited for the study through the national Stalking Support Centre for victims and perpetrators of stalking. The experts on violence at the Centre contacted families meeting the following criteria at the time of recruitment: (1) The family’s clienthood had been prolonged due to the father’s stalking, (2) the child was or had been a client in their own right, and (3) the child was otherwise suitable as a participant in light of the family situation or the child’s age and stage of traumatisation. All the children were willing to participate in the study, but a number of them were excluded on the grounds that the experts deemed it too risky for them to participate because the father’s stalking behavior was considered too intense, or the mother and the children were planning to relocate in the near future. The total sample consisted of children who passed the above-mentioned criteria. The children did not have diagnoses that would have affected participation in the study. Had there been more children or young people passing the criteria, they would have been included in the sample.

The data were collected using interviews and therapeutic action group sessions with the children. The researchers used different methods because the aim was to collect data from children who are hard to reach and whose life situations vary, as the intensity of father’s stalking behavior can vary over time (Logan & Walker, 2017). Also, the aim was to reach both small children and young people, who may prefer different data collection methods. The data collection was planned in collaboration with the experts on violence with the objective of identifying procedures that would suit each child’s age and life situation and best protect and strengthen their wellbeing. The two methods enabled the collection of data in different forms: through action, interaction, and oral and written output. The experts saw that some children could express their experiences verbally in interviews, while others needed different methods to support their narration and to benefit from a therapeutic group process with peers. The experts on violence collected the research data periodically between 2012 and 2019.

In the first set of data, 18 children and young people (15 girls and 3 boys) aged between 4 and 21 participated in interviews. The children could choose whether to be interviewed alone or with their sibling. Six of the children were interviewed individually and the rest of them in pairs. The interviews were based on teller-focused interviewing (Hydén, 2014) and open narrative methodology. All interviews shared the same thematic framework, but it was adapted to the age and developmental stage of the child to gain a child-friendly atmosphere (Eriksson & Näsman, 2012). The themes discussed were actions of stalking, experiences of violence, intimate relations and emotions, supporting resources, need for support, and experiences of encounters with professionals. The children were informed that they can talk freely about their experiences and the interviewers followed up with questions. The experts also encouraged the children to talk by giving positive feedback and showing that they valued their descriptions. The duration of the interviews varied between 18 and 105 min. They were recorded and transcribed verbatim.

The second set of data derives from three therapeutic action groups focusing on the children’s experiences of the conflicted separation of their parents. Altogether 13 children (8 girls and 5 boys) aged 2–12 from seven families participated in the groups. The children were gathered into groups of three to five based on their sibling relations and experiences. Each group had from eight to ten sessions and they met once a week, comprising 28 sessions in all. The group process was planned in such a way that each session was based on one or two themes and related activities. The themes concerned the children’s close relations, parents’ separation and violence, (in)security, emotions, and supporting resources. The activities in the sessions included drawing, painting, discussion, making a collage, and listening to music, stories and drama. The duration of each session was 90 min and they were videotaped. The group process consisting of multiple sessions made it possible to gain in-depth data on the children’s experiences in an ethically sound way and gave the children an opportunity to feel secure and confident both physically and emotionally so that they could disclose their experiences. The creative and embodied activities were used to offer channels for verbal disclosure, as experiences of stalking can be difficult to articulate for some children (see also Fellin et al., 2019). The group sessions were not transcribed, as they could not be adequately documented because of the action and simultaneous talk in them.

Throughout the research process the ethical requirements of sensitive research were considered, and the study committed to the ethical guidelines of The Finnish National Board on Research Integrity (2019). The research project was approved by the Research Ethics Committee of the University of Lapland. The two local collaborating chapters of the Stalking Support Centre signed the research permit. The children and their guardians gave their informed consent, but in the case of a two-year-old participant, the parents gave their consent on behalf of the child. Lastly, all the children had the possibility to withdraw from the study during any phase.

### Data Analysis

The data analysis was content-oriented (Gibbs, 2007/2009). In the first phase it concentrated on how the children described their family relations and other relations, how they acted within those relations, and how they described the connections of these relations to parental stalking. The answers were sought through data-based reading of the transcribed interviews and by watching and listening to the videotaped group sessions. The close relations with which the children associated their experiences of parental stalking concerned their fathers, mothers, siblings, friends, relatives, and pets. The children described these relationships in terms of the roles of family members, including the expectations, duties and responsibilities these roles entail, and depicted the emotions and thoughts arising from the family situation burdened by stalking. The second phase included an interpretation of both sets of data, where the interest was in the children’s sense of belonging in close relations. The aim was to find out how the children constructed their sense of belonging and their perceptions of who they belong with. On the basis of the analysis, four dimensions of children’s sense of belonging in family relations were identified: (1) varying belonging, (2) distancing belonging, (3) non-belonging and (4) holding belonging. Throughout the analysis, it was important to be aware of potential biases in the interpretation of the children’s voices concerning their family relations, as adult listeners’ understanding of children’s accounts may vary greatly. Children may have their own semantic understandings, and there is as risk that researchers reify children’s voices by applying their own interpretive frameworks to them (see also Spyrou, 2011). Next, the dimensions of children’s sense of belonging will be elaborated on and discussed in relation to previous studies. The children’s age, gender, and other identifying characteristics in the quotations are anonymized to protect their privacy.

## The Four Dimensions of Children’s Sense of Belonging

### Varying Belonging

Children’s varying sense of belonging becomes visible in their relations towards their abusive fathers. It is based on a child–father relationship characterized by the child’s ambivalent feelings towards the father, and as a result of this ambivalence, the sense of belonging or not belonging with the father may change according to the situation. This dimension of belonging is prevalent among younger children in particular, but also visible in older children’s experiences. Children’s varying sense of belonging originates in the good and bad sides of the father, but also in children’s loyalty to their parents and in their perceptions of the ideal family. There are several examples in the data where children describe their positive views and memories of the father:

Yes, I remember that when I was small, we did a lot of stuff together and I have good memories, too, but maybe the bad ones are uppermost in my mind. … Or, well, we do have a lot of photos where I for example sleep on my dad’s belly. (Child)

For the most part, the children indicated having difficulties in maintaining a relationship with their father. One of the tasks in the group sessions was to create, by means of bricolage, a tree of social relations where apples represented the children’s good relations and lemons stood for difficult ones. In making the tree, some children showed ambivalent feelings towards their fathers, as reflected on by one child in trying to decide whether the father would be best represented by an apple or a lemon:

There has been some apple to it, too, sometimes [thinking of father’s good sides], but I still won’t put an apple there… That lemon [father’s bad sides] is actually my father. The one I have to meet… and then I should discuss [with father]. I actually go to meet him but... well, I do go to meet him, but I just tell him directly what I think because he won’t believe otherwise.

The group process revealed that a child’s conception of their relationship with the father may change from one session to another. During the next session, the child quoted above attached an apple next to the lemon, saying that the father also has good sides. The task also shows that children living in the same family can have different feelings towards and contradictory relationships with their father. In one family, all siblings of the family had supervised meetings with their father. In the tree of social relations, the older siblings represented the father as an apple while the youngest sibling saw that the father was dead.

The data show that children’s perceptions of their father’s having good and bad sides and their incomplete knowledge of the family situation may make them want to maintain their relationship with the father (also Cater, 2007). For example, in one of the group sessions, one child expressed a longing for the father and reported having difficulties in understanding the restrictions on meeting him. One of the interviewed children wanted to rebuild a relationship with the father after a break in meetings with him. Often the children acknowledged their father’s violent behavior as a part of his multifaceted personality, where good memories neutralized bad behavior and distanced violence from the father’s relative goodness (also Callaghan et al., 2015; Cater, 2007).

In varying belonging, the children expressed an emotional need to belong with their father, but the father’s behavior conflicted with their desires and made it difficult for them to belong with him in a reciprocal manner. The children’s loyalty to their parents may have affected the way in which they perceived their relation to the father. In the interviews, two children reflected on their own and their siblings’ relationship with their father:

He is anyway my father. (Child).

But I think my [sibling] still wants some kind of relationship [with dad]. My [sibling] really tries to get his attention and that kind of care that my [sibling] didn’t get from him. And they don’t have a good relationship. My [sibling] recognizes it and always says that he [father] isn’t a good person. But still my [sibling] wants to be in contact with him. (Child)

The varying sense of belonging shows that children have diverse experiences of their relationships with their fathers. Children may, for example, seek to reinforce their relationship with the father even though they may simultaneously tacitly question it. This, in turn, may hinder children’s possibilities to define their family relations based on subjective experiences of their quality. This is indicative of children’s simultaneous need of access to and protection from their abusive parent (Cater, 2007).

### Distancing Belonging

The dimension of distancing belonging concerns children who have started to distance themselves from their fathers rather than having a unified sense of belonging to the family. The interviews and group sessions indicate that the children questioned their belonging with the father because the father’s behavior caused disappointment, insecurity and fear in them. In one of the interviews, the interviewer asked a child what had happened next when the child had started to take distance from the father already when the family were still living together:

We didn’t talk much anyway, or he doesn’t seek contact in a way that a child would want… I didn’t ask anything from him, I totally ignored him. If I had something to say I went straight to my mum… I didn’t agree to go anywhere where the whole family was going if he was coming with us… I started giving quite clear signs that I don’t want him around me and I don’t want him to talk to me. I can’t stand his presence.

The children were distressed and perceived their father’s stalking of the mother and/or themselves as violent. Particularly older children were aware of the fact that their father’s behavior was culturally unacceptable and had become critical of him. They were able to form their own perceptions of him (also Elklit et al., 2019; Krampe, 2003) and evaluated the negative consequences of stalking for themselves, their mother and their siblings.

- Do you have meetings with your dad these days?
- My younger siblings meet him. I don’t.
- Why don’t you?
- I don’t want to. ….
- Was there something about the meetings that wasn’t nice?
- They were distressing.
- Was there anything good about them?
- No.

The children’s sense of belonging with their father started to erode also because the father exhibited inconsistent behavior alternating between caring and threatening behaviors (also Callaghan et al., 2015; Cater, 2007). The children were aware of the father’s subtle ways, such as tempting the child into his car with candy, to exert control over the children and/or mothers (also Callaghan et al., 2015). The children realised their instrumental role and that contacts with their father had nothing to do with his missing them, but rather with his interest in the mother.

Back then he still tried to get in contact with us children, or actually to get in contact with mum through us [children]. He sent messages to us too, and went like, let me know how mum is doing, and, you can get 10 euros if you send me a photo of mum. (Child)

Within the dimension of distancing belonging, the children constructed their sense of belonging based on self-identification and other forms of identification (Yuval-Davis, 2011). The latter came into play where the father or other people – particularly relatives and professionals – sought to influence the children’s understanding of their sense of belonging with the father (see Konstantoni, 2012; Krampe, 2003). Some of the children got limited support from others to define independently who belongs to their family (also Staf & Almqvist, 2015; Swanston et al., 2014) and saw that authorities, or other adults, cannot force them to be in contact with the father if they do not want to. Some children felt confused when the father himself or relatives on the father’s side maintained the image of a caring father:

Well, how can you think of your dad in that way, he [father] said. (Child)

He appealed [to the fact] that I’m his biological child and we should be in contact because of it. Suddenly, I was dear to him. (Child)

According to them [relatives on the father’s side], dad has done nothing, or nothing like that. They didn’t believe that, so they blamed our mum for those things and then relations between them broke down. (Child)

In distancing belonging, the father’s abusive behavior forced the children into a conflict situation where they had to consider how to position themselves in relation to the family members and how to make sense of their belonging. The children were able to construct their own understanding of the family relations, which created the ground for establishing new positive social relations and strengthening existing ones. The lack of genuine interest on the part of the father undermined the children’s sense of belonging with him (Giddens, 1991; Jamieson, 2011).

### Non-belonging

As a consequence of the father’s stalking behavior, some children had developed a sense of not belonging with their fathers that sometimes extended to relatives on the fathers’ side. This was particularly prevalent among older children, but also among young children, such as one 6-year old child. The children’s sense of not belonging with their father illustrates their ability to view their family relations critically. One child did not have a relationship with the father and did not want to be in contact with him:

- What do you think, what kind of a relationship do you have with your father?
- I don’t really have a relationship with him.
- Would you like to describe it or give some details about it?
- After mother and father separated, in the beginning he sent messages and called [me] on the phone and stuff. I blocked him because I didn’t want to be in contact with him.

The children had a good understanding of how the father’s controlling dynamics operated within the family relations and they brought up both the father’s violent behavior towards the mother and/or themselves and his contact attempts, which were not considered sincerely caring (also Callaghan et al., 2015). Some children stated that the main reason they did not want to see their father was the poor quality of the child–father relationship, and many children said that their father was not as present as he should be when spending time with children (also Cater & Forssell, 2014; Holt, 2015; Pang & Thomas, 2020). Thus, if the children’s relation to the father was not mutually rewarding, some of them eventually broke off the relation (also Øverlien, 2013; Staf & Almqvist, 2015). In an interview, one child said that the younger siblings did not want to relate to the father because of his poor fathering skills:

They [the younger siblings] don’t want to see him. None of them can really relate to him. And they are shy with him because he never really built any kind of relationship with my [younger sibling], and when mum and dad separated [the sibling] was seven or six years old. One day [the sibling] said that “He [dad] never talked to me, I don’t even know the person, so why bother to go and meet him again.” None of them has expressed a need to see him. They are rather happy that we have got rid of him.

Some of the children were aware of the father’s manipulative role in the family and his attempts to affect the children’s thinking and action, for example, by disparaging the mother, blaming her or the children for the family situation, and treating the siblings in an unequal manner by favouring one over the other. They found their father’s behavior immoral and wrong, as narrated by one child in an interview:

It has been a relief for me in the sense that I have been able to set boundaries. And to decide that I don’t want to be in contact with him because I saw how he treated and still is treating my other siblings who are in contact with him. Or how he used to treat my mum, and I didn’t want to be part of that. Because when we were still in touch he could use me against the others without me knowing it. So that he manipulated people.

The cultural and societal context also became visible in the way the children gave reasons for their sense of not belonging with their fathers. Education and the children’s awareness of their own rights as citizens were important factors in supporting the children in making decisions concerning their family relations:

- How did you know that a 12-year old can make the decision independently [to decide not to be in contact with the father]?
- Then you have quite a lot of rights.
- Yes, that’s true. How did you know that a 12-year old…?
- From the social studies book [a school textbook].
- So, you learned at school, in social studies class, that when a person is 12 years old, they can decide whether they go [to meet the father], and you said that to the supervisor at the meeting?
- Mmmh. Yes, we have been taught that the rights, you have more rights when you turn 12.

The children also contradicted the positive cultural understanding of the child–parent relationship. The contradiction occurred when, for example, some children expressed positive emotions when they talked about the parents’ separation or the father’s criminal sanctions. One child wished that the father would be sentenced to prison or even die. The difference between established cultural codes and the children’s actual experiences became visible especially when the friends of one of the children expressed their condolences over the death of the child’s father, although this was a relief for the child:

We had a friend visiting us then and [the friend] just watched, confused by my reaction [Joy and happiness at the father’s passing away].

The children experiencing a sense of non-belonging were aware of the power and control they can have in relation to their father, and this awareness produced a strong sense of self-reliance and confidence in their own ability to affect their family relations (also Callaghan et al., 2015). The children’s sense of belonging, or the lack of it, was self-identified (Yuval-Davis, 2011) and they knew that they are entitled to decide with whom they want to belong. The children’s psychological relation to their father can be described as one where he is an ‘anti-father’; that is, the children deny their need for a father and their need to maintain a sense of belonging, a reaction which is based on fear or antagonism (Krampe, 2003). Non-belonging acknowledges that children can be active in searching for belonging that enhances their wellbeing and enables them to find moral significance in their social relations.

### Holding Belonging

Holding belonging can be regarded as the core of children’s sense of belonging in that it enables them to maintain reciprocal and rewarding social relations. In holding belonging, children’s significant others include persons who provide care, comfort and security. In the group session during which the participants made, by means of bricolage, a tree of social relations where apples represented positive relationships, most of the children ascribed apples to the mother, friends, relatives and pets. Also, in the interviews, the children described how the aforementioned people and pets were important in providing support and security.

– Who are your important persons at the moment?

– Mum. And the other family [members] too. Friends. Or I have a quite close group of friends at the moment. People I have known for many years and they know about my things and I know a lot about their things. There are others, too, close relatives with whom I’m more in touch.

– Did it [the dog] make you feel safe?

– Very much. It let me know if something was happening; it barked when it heard noise in the yard. And for sure, if I had let it loose and if it had attacked dad, no one would dare to come. Yes, it protected me a lot that way.

The basis of children’s holding belonging is having relationships with persons who can attend to their emotional needs and provide safety (also Leung, 2015; Noble-Carr et al., 2020). The children shared negative emotions, such as fear and disappointment, with their mother, but also positive ones, such as feelings of trust and care. It was crucial for the children that also the mother and siblings felt safe. Some children adopted a supportive role in their relationship with the mother and siblings by taking care of and protecting them from the father’s stalking. This ‘controlling care-giving’ behavior can be a way for the child to have their need for closeness met (Staf & Almqvist, 2015), even though it was also a major burden for many children. For example, in the group sessions, one child constantly looked after the younger sibling, while in the interviews, some children said that they take care of their mother and siblings. This behavior breaks the social premise concerning the roles of family members.

In creating holding belonging, the way in which children and other people, such as professionals, understand family as a cultural concept plays a crucial role. For the children, it was important that professionals and other adults identify with their lived experiences of family relationships and separate their experiences from the general discourse of the family unit. One child reported that it was not possible to talk about a ‘real’ family until the parents had separated and the child moved away from the father:

At least in the beginning it was safe to be at home. I don’t know, but the atmosphere was such that you didn’t have to be alert. You really could be at home and do what you wanted…. I don’t know, maybe it just was more like being a family. We started to do more things together, which everyone was happy with…. I guess. Maybe it just kind of is. A family. Maybe it makes [me] happy in a different way than it used to.

The different cultural understandings of family also came forth when discussing what has been helpful for the children and how the service system could be developed. The children considered it important that they can discuss their family situation particularly with mother and siblings, but also with professionals. One child proposed that professionals could appreciate children’s own views of belonging more and thereby support the relations that children themselves consider as a resource:

I would say that you wouldn’t necessarily support the family’s staying together, but you could rather support the children’s wellbeing. They [professionals] don’t always... home is not always the best place to be. Maybe it is also about children wanting to protect their parents. (…) Or the child may feel guilty if he/she talks against a parent or tells others what [the parent] has done and [the child] ends up saying that [he/she] does not want to be separated from [the parent].

The value of holding belonging lies in its pervasiveness; it must not have any boundaries that force children to shape their sense of belonging. This freedom follows from children’s and human rights (see Yuval-Davis, 2011), which entitle children to live meaningful lives. Holding belonging enables children to create pure relationships (Giddens, 1991) and carry out practices that satisfy their need for intimacy, connectedness and being attuned to each other (Jamieson, 2011) under the threat of stalking. It promotes children’s ontological security (Giddens, 1991) and is a prerequisite for their coping.

## Acknowledging Children’s Sense of Belonging in Post-Separation Parental Stalking

**Fig. 1:**
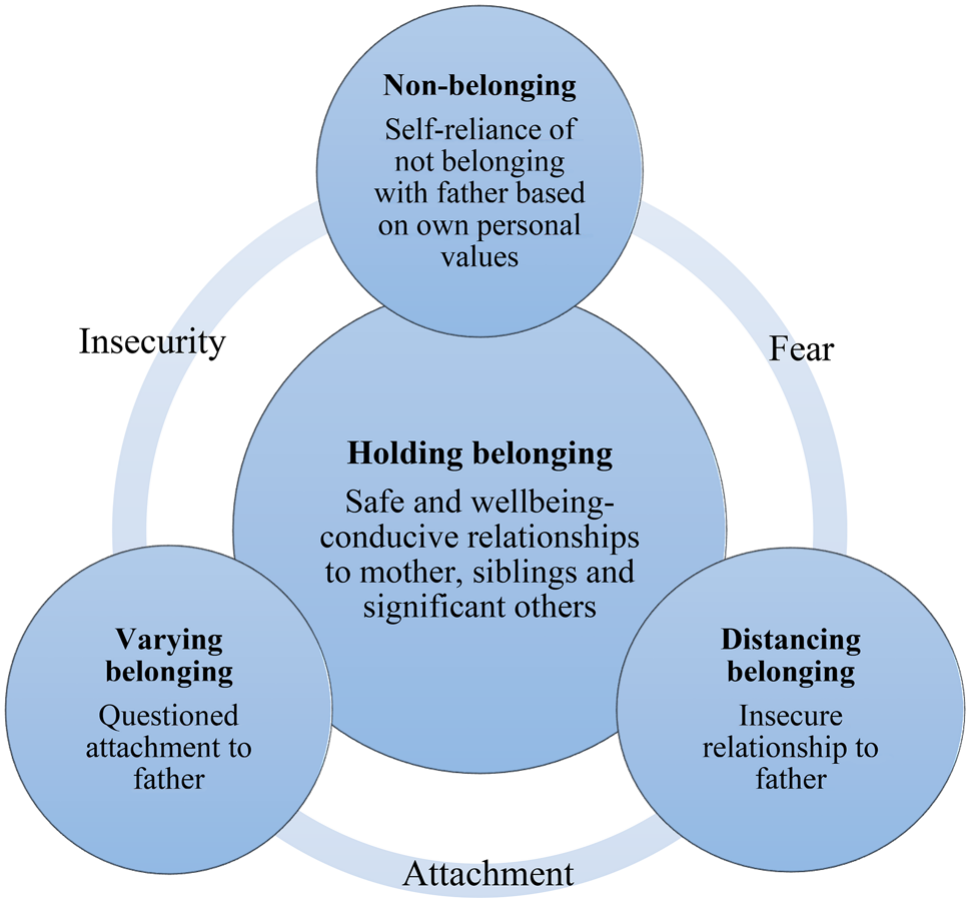
Children’s sense of belonging in the context of post-separation parental stalking

The dimensions of children’s sense of belonging – varying belonging, distancing belonging, non-belonging and holding belonging – are parallel and not mutually exclusive. According to the qualitative data analysis, children’s sense of belonging is mainly determined, on the one hand, by fear and insecurity and, on the other hand, by attachment and safety related to the family members and the way in which this correlates with children’s personal values. The dimensions of belonging are illustrated in Fig. 1.

The findings show that children’s sense of varying belonging, distancing belonging, and non-belonging is constructed primarily in relation to their abusive father. Consequently, children question their belonging with the father to different extents. A sense of varying belonging does not enable children to set boundaries or exclude the persons who harm them or jeopardize their wellbeing (see Yuval-Davis, 2011), but children do start questioning their attachment to the father. However, children may still have an emotional need to belong with the father (Cater, 2007). As for distancing belonging, children are capable of constructing a personal understanding of their family relations and questioning their relationship with the father, identifying it as a source of insecurity. When children experience non-belonging with their father, their immediate social context becomes less comfortable and they no longer feel that they ‘fit’ with all of their family relations (May, 2011). Children are very clear about the need to exclude the father from their own and their mothers’ lives (also Øverlien, 2013). Children’s sense of not belonging with the father appears as a positive experience (May, 2011) that allows them to reflexively control their familial relations and entitles them to fully define their belonging (Giddens, 1991). Contrarily, holding belonging relates to persons with whom children feel belongingness and who can provide emotional and cognitive reciprocity and satisfaction (Jamieson, 2011). Holding belonging represents a stable element in children’s experiences, whereas the other dimensions are in flux and depend on the child’s age and the particular time and place (May, 2011; Yuval-Davis, 2011).

The findings of the study echo the earlier studies on parental stalking and coercive control in terms of the ways in which fathers use children as a means of contacting the ex-partner. The ways in which fathers manipulate children and coerce and control the everyday lives of children and their mothers (e.g., Nikupeteri & Laitinen, 2015; Callaghan et al., 2015; Clements et al., 2021; Elklit et al., 2019; Løkkegaard et al., 2019) may prompt children to distance themselves from their abusive father and to seek comfort and support from their abused mother and siblings (e.g., Katz, 2015; Lapierre et al., 2018). Moreover, the study shows that children in the same family do not necessarily share the same sense of belonging (also Swanston et al., 2014) and that their sense of belonging may fluctuate. This emphasizes the significance of the fact that professionals understand children’s family relations and sense of belonging as a multifaceted issue rather than think that children’s family relations have a uniform influence on all family members (also Angel, 2014).

In addition, the findings show that even though children can, to a certain extent, choose with whom and to what they want to belong (Angel, 2014), they may not have a say in situations where professionals assess that contact between a child and his or her father is in the child’s best interest, even if the contact poses a risk of continued violence against the child and mother (Nikupeteri & Laitinen, 2015; Bruno, 2018; Radford & Hester, 2015; Staf & Almqvist, 2015; Tisdall et al., 2021). Children’s sense of not belonging with a stalking father challenges the ideal of joint parenting and children’s continued contact with both parents after the parents’ separation (also Elizabeth et al., 2012; Jaffe et al., 2009; Radford & Hester, 2015) by adding the child’s personal experience of belonging to the evaluation of the quality of family relations. Taking note of the child’s experience matters, as it can reduce the identified risk of a child or the mother being murdered after separation in the course of custody disputes and contact arrangements (Jaffe et al., 2009; Spearman et al., 2022). For example, the Center for Judicial Excellence (2022) in the United States has tracked the number of children murdered by a parent when divorce, separation, custody, visitation or child support was mentioned in news coverage. Since 2008, 871 children have been murdered by a divorcing/separating parent. System failures were reported in 118 cases, where the killing of a child would have been preventable.

Disclosing children’s sense of belonging requires that professionals work with children for long periods and acknowledge their multifaceted sense of belonging in family relations. Also, children’s statements of not belonging with the father should be taken seriously (also Bruno, 2018) and as ‘real views’ of children (Tisdall et al., 2021). A nuanced understanding of children’s sense of belonging enables professionals within social and health care services and law enforcement to place value on children’s views of post-separation family relations that provide protection, are mutually rewarding and increase their wellbeing (Jamieson, 2011). Strengthening children’s social identity with trusted adults, siblings and peers makes it possible for them to experience secure and self-determined belonging (Konstantoni, 2012).

There are some limitations concerning the study. Owing to the small amount of data and the wide age range of children (2–21 years) involving multiple developmental stages, it was not possible to focus on the sense of belonging of a certain group of children. It can be anticipated that experiences of family relations vary significantly between pre-schoolers and children in their late teens who have more legal rights to manage their own lives. Also, had the sample included more boys, the analysis could have covered the role of gender in children’s experiences. In particular, analyzing the therapeutic action group entailed a risk of over- or misinterpreting the children’s experiences, but analyzing the data by a group of researchers reduced this risk to some extent. Given the rather narrow focus of the present study involving children’s immediate family relations, a more comprehensive understanding of children’s sense of belonging in abusive post-separation family contexts could be developed by studying the role of friends, relatives and pets in constituting a sense of belonging. Moreover, as environmental aspects play a crucial role in one’s sense of belonging, and as our data and analysis are limited to studying how for example relocation to a new area or school affects a child’s sense of belonging, environmental factors call for further investigation.

To our knowledge, no prior studies have examined children’s sense of belonging in the context of stalking and domestic violence. Our study shows that investigating children’s sense of belonging can bring added value to previous studies on the quality of children’s relationships with an abusive and abused parent and on motherhood and fatherhood when one parent abuses the other and/or the children (e.g., Cater & Forssell, 2014; Katz, 2015; Lapierre et al., 2018; Staf & Almqvist, 2015). Belonging enables the examination of the multifaceted nature of children’s family relations embedded in the broader sociocultural context, also considering children’s emotions, personal values and moral views characterizing their family relations (see May, 2011). In terms of future research, belonging can be seen as a theoretical framework applicable to analyzing children’s experiences of different forms of violence in families.

## Conclusion

According to the findings of the study, professionals working with children experiencing violent post-separation family situations should acknowledge children’s capacity to analyse the quality of their family relations and to assess who they belong with. It is essential that professionals let children form family and other social relations according to their subjective experiences and that they support those relations (also Noble-Carr et al., 2020). A finer-grained understanding of children’s sense of belonging is needed in law enforcement and social and health care services so that children’s safety and best interest can be guaranteed when faced with domestic violence and stalking. The findings are applicable particularly to cultures that share the Western ideal of the continued involvement of both parents in children’s post-separation lives.

## Footnotes

1 The interviewed woman and her child are part of our research project Children's Knowing Agency in Private, Multiprofessional and Societal Settings - The Case of Parental Stalking.
